# Sliding Water Droplet on Oil Impregnated Surface and Dust Particle Mitigation

**DOI:** 10.3390/molecules26040789

**Published:** 2021-02-03

**Authors:** Saeed Bahatab, Bekir Sami Yilbas, Abba Abdulhamid Abubakar, Ghassan Hassan, Anwaruddin Siddiqui Mohammed, Hussain Al-Qahtani, Ahmet Z. Sahin, Abdullah Al-Sharafi

**Affiliations:** 1 Mechanical Engineering Department, King Fahd University of Petroleum and Minerals (KFUPM), Dhahran 31261, Saudi Arabia; g201074560@kfupm.edu.sa (S.B.); abba.abubakar@kfupm.edu.sa (A.A.A.); engghhh08@gmail.com (G.H.); g201709110@kfupm.edu.sa (A.S.M.); qahtanih@kfupm.edu.sa (H.A.-Q.); azsahin@kfupm.edu.sa (A.Z.S.); alsharafi@kfupm.edu.sa (A.A.-S.); 2 Center of Research Excellence in Renewable Energy (CoRE-RE), King Fahd University of Petroleum and Minerals (KFUPM), Dhahran 31261, Saudi Arabia; 3 Energy Research & Innovation Center (K.A.CARE), Dhahran 31261, Saudi Arabia

**Keywords:** water droplet, silicon oil impregnation, crystallization, hydrophobic, polycarbonate

## Abstract

Self-cleaning of surfaces becomes challenging for energy harvesting devices because of the requirements of high optical transmittance of device surfaces. Surface texturing towards hydrophobizing can improve the self-cleaning ability of surfaces, yet lowers the optical transmittance. Introducing optical matching fluid, such as silicon oil, over the hydrophobized surface improves the optical transmittance. However, self-cleaning ability, such as dust mitigation, of the oil-impregnated hydrophobic surfaces needs to be investigated. Hence, solution crystallization of the polycarbonate surface towards creating hydrophobic texture is considered and silicon oil impregnation of the crystallized surface is explored for improved optical transmittance and self-cleaning ability. The condition for silicon oil spreading over the solution treated surface is assessed and silicon oil and water infusions on the dust particles are evaluated. The movement of the water droplet over the silicon oil-impregnated sample is examined utilizing the high-speed facility and the tracker program. The effect of oil film thickness and the tilting angle of the surface on the sliding droplet velocity is estimated for two droplet volumes. The mechanism for the dust particle mitigation from the oil film surface by the sliding water droplet is analyzed. The findings reveal that silicon oil impregnation of the crystallized sample surface improves the optical transmittance significantly. The sliding velocity of the water droplet over the thick film (~700 µm) remains higher than that of the small thickness oil film (~50 µm), which is attributed to the large interfacial resistance created between the moving droplet and the oil on the crystallized surface. The environmental dust particles can be mitigated from the oil film surface by the sliding water droplet. The droplet fluid infusion over the dust particle enables to reorient the particle inside the droplet fluid. As the dust particle settles at the trailing edge of the droplet, the sliding velocity decays on the oil-impregnated sample.

## 1. Introduction

Mitigating dust from surfaces in outdoor environments is challenging since dust strongly pins over the exposed surfaces [1]. Change in climate triggers a dust storm, which causes several problems to be tackled in many sectors such as energy [2], medical [3], and agriculture [4]. In renewable energy and transportation sectors, dust settlement has an unfavorable effect on the system performance because of the reduced system output, which relies on the optical transmittance of solar energy devices and detectors. In solar energy applications, photovoltaic panel efficiency is related to the amount of solar radiation reaching the panel’s active surface. Similarly, for autonomous transportation systems, proper transmittance and detection of the signals from the environment become vital for sustainable operations; however, dust settlement on surfaces creates obstacles for the incoming signal to be received by the detection system. Moreover, different techniques have been purposed to mitigate the dust from surfaces. Water splashing [5], air-jet blowing [6], mechanical brushing [7], electrostatic repelling [1], and self-cleaning [8] are some of the dust mitigation methods. As the optical transmittance becomes important such as PV panels, the regular cleaning of dusty surfaces becomes essential. Moreover, one of the techniques to lower dust adhesion is to lower the dust contact area on the settled surface. This can be realized via texturing of surfaces; hence, interfacial contact between dust and surface is reduced by the trapped air in the texture gaps. Depending on the surface texture topology, the wetting of the surface changes towards the hydrophobic state. This further lowers the pinning force of dust on surfaces [9]. Although the surface having a hierarchical texture of micro/nanopillars gives rise to hydrophobic wetting, the texture of the surface results in scattering and diffusion of incident optical rays. This reduces the optical transmittance of the hydrophobized samples. To enhance the UV visible transmittance, thin oil film matching the refractive index of the textured surface can be utilized via spreading over the textured surface [10]. However, the spreading of oil film over the sample surface is related to the spreading factor, which is governed by the surface tension of oil, interfacial resistance, and surface free energy of the textured surface [11]. Hence, the minimum thickness of the film remains critical in terms of the formation of a continuous oil film on the surface. As the dust grains settle over the oil film, the oil infusion takes place on the particle surfaces. This makes it challenging towards mitigating the settled particles from the oil film surface. Consequently, the examination of optically transparent textured surfaces and thin oil film impregnation towards dust particles mitigation from the textured surface becomes necessary.

Considerable research studies are carried out for exploring the self-cleaning of surfaces. Hydrophobic and superhydrophobic surfaces remain favorable for self-cleaning applications. Surface free energy of the textured material is one of the key parameters achieving the superhydrophobic state on the textured surface. Hence, textured surfaces can be coated by a low surface energy material, such as octyltriethoxysilane (OTES), in the secondary process. Nevertheless, the durability of such a coating is challenging in outdoor environments [12]. Besides, applying octadecyl isocyanate (ODI) on TiO_2_ particles can create similar wetting states on the surface by creating hydrophobic alkyl chains with the hierarchical crystalline structure [13]. The coating resulted can undergo hydrogen bonding among adjacent alkyl chains, which greatly increases the sustainability of the coating surface. Introducing nano-composite thin films on surfaces via depositing silica nanotubes can improve the hydrophobic state of the surface and the optical transmittance of the surface may reach up to 87% [14], which is considered to be low for photovoltaic applications. Tailoring the surface texture for the improved hydrophobic wetting state is also possible via lignocellulose coating [15]; however, aging of the coating in harsh environments requires further investigations. In addition, coating of surfaces by perfluoroalkylated laponite nanoparticles can create hydrophobic and omniphobic surfaces [16]; yet the contact angle for water droplet is about 106°, which limits the self-cleaning application of the coated surface by a rolling water droplet. Antifriction coating of surfaces can minimize the damages of hard particles, such as scratches created by the dust particles [17]. Although surface lubrication can improve the scratch resistance, the UV visible transmittance can be reduced considerably for energy harvesting applications. The liquid-infused porous surfaces can also be utilized for self-cleaning applications. A typical surface can be formed via the infusion of silicon oil on the porous electropolymerized polyaniline surface [18]. Care must be taken towards covering the textured porous surface with a uniform film thickness of silicon oil. Besides, the optical transmittance of the silicon oil-impregnated surface remains vital for the self-cleaning of optically transparent surfaces. The oil refractive index matches with the refractive index of the impregnated surfaces, the optical transmittance of the oil-impregnated substrate improves [10]. One of the applications of oil impregnation of the transparent surfaces is to enhance the UV visible transmittance of the hydrophobic samples; in which case, the optically transparent and oil-impregnated surface can be utilized for self-cleaning applications [19].

On the other hand, environmental dust has various densities because of variation in composition [20]. Due to the non-stochiometric composition of constituting compounds of some small size particles, these particles adhere strongly on solid surfaces even though the surface is being hydrophobized [21]. In addition, several coating techniques have been developed towards hydrophobized surfaces for self-cleaning applications [14,17,22], yet further tests are needed for the assessment of dust adhesion and mitigation from these surfaces. Dust mitigation from hydrophobic surfaces via utilizing gravitational potential, by surface tilting, may not be always successful and some dust residues remain on the surface. Introducing the avalanche effect on dusty surfaces can improve the dust mitigation rates, particularly, for hydrophobic surfaces; however, creating the avalanche effect becomes challenging on dusty surfaces [23]. Moreover, chemically modification of the dust particles by diluted hydrofluoric acid lowers dust adhesion, which enhances dust mitigation from surfaces [9]; nevertheless, the process requires additional cost for dust treatment. Hence, the dust mitigation incorporating the oil film on optically transparent surfaces may become fruitful in terms of the effective self-cleaning process [10,19]. In addition, optical transmittance remains important for surfaces, which are used for energy harvesting such as photovoltaic panel surfaces. Hydrophobizing surfaces towards reducing dust adhesion create surface textures, which diffuse, scatter, and partially absorb the incident optical radiation while reducing the optical transmittance. The use of optically matching fluids, such as oils, with the textured surface, can improve the optical transmittance. In general, towards the self-cleaning application of an oil-impregnated surface, a water droplet is used and the sliding velocity of the droplet over the oil film plays a major role in the mitigation of particles from the surface. The sliding velocity depends on the shear rates across the water-oil boundary, the surface tilting angle, and the volume of the droplet volume. The particle mitigation from such surfaces also depends on the water and oil infusions over the particle surface. As the water infusion height becomes larger than the oil infusion height on the particle, the particle most likely can be taken by the sliding droplet fluid. Therefore, the investigation of particle mitigation from the oil-impregnated optically transparent surface becomes essential. Although oil impregnation and optical transmittance of the impregnated surface were studied before [10], the sliding droplet dynamics and the mechanism of dust mitigation from oil impregnated surface by sliding water droplet was left for future study. Consequently, in the present study, sliding droplet behavior over the silicon oil film, which is located on the crystallized polycarbonate surface, is examined, and the mechanism of dust removal by sliding water droplets is explored. The crystallization of polycarbonate surfaces is carried out by adopting the solution treatment method. The spreading factor of silicon on crystallized polycarbonate surface is evaluated and silicon oil infusion over the dust particles is assessed via accommodating the high-speed records and the tracker program. The influence of dust particle orientation and the thickness of the film on the particle mitigation mechanisms is further examined experimentally. The sliding velocity of the water droplet on the silicon oil-impregnated surface is assessed through the energy balance equation and the resulting droplet sliding velocity is compared with that obtained from the experiments. The optical transmittance of the surface before and after silicon oil impregnation is also evaluated.

## 2. Experimental

Table 1 gives the experimental parameters used in hydrophobizing of polycarbonate samples and the dust mitigation from the oil-impregnated sample surfaces. The samples were prepared from p-hydroxyphenyl (polycarbonate). Acetone solution with 30% concentration (by volume) was used to crystallized the sample surfaces. The immersion duration of samples in acetone solution was set at 4 min in line with the early work [24]. The contact angle of the crystallized surface was evaluated using a Goniometer (Kyowa, Niiza, Japan, model—DM 501) while adopting the high-precision measurements and analysis (HPDSA) [25]. Since the crystallized surface has textures composing of micro/nano spherules and fibrils, the optical transmittance of the textured samples was evaluated using the UV visible spectrometer (67 Series spectrophotometer, Jenway, Chelmsford, UK). The topology and surface texture were analyzed utilizing electron scanning (Jeol 6460 electron microscope), optical, and atomic force (Flex-Axiom, Nanosurf, Liestal, Switzerland) microscopes. Since the optical transmittance was found to be reduced considerably after crystallization of the polycarbonate surface, due to surface reflection and scattering of the incident optical radiation from the texture morphology, silicon oil was introduced on the sample surface to enhance the UV visible rays transmittance. Silicon oil impregnation of the surfaces was conducted in line with the early work [10]. In this case, silicon oil with viscosity 10 cst, density 935 kg/m^3^, and surface tension 35 mN/m were utilized impregnating the sample surfaces, and care was taken to cover the entire sample surface. The thickness of the oil film was evaluated via an ellipsometer (M-2000 J.A. Woolam Co., Lincoln, NE, USA) after impregnating the sample surfaces. The ellipsometer measurements depended on the monochromatic light polarization over the oil surface. Two oil film thicknesses were incorporated in the experimental study, which were 700 µm and 50 µm, and the roughness of the oil film was estimated as 0.42 nm. This arrangement provides to examine the influence of oil film thickness on the water droplet movement on the impregnated surface. The high-speed records utilizing Dantec Dynamics SpeedSense 9040 and tracker program were used to record and evaluate droplet velocity on the inclined impregnated sample surface. The droplet data recording by the high speed facility was carried out at 5000 fps and the system had a resolution of 1280 × 800 pixels with the size of 14 µm × 14 µm. The repetitive records for the droplet motion under the same conditions were used to determine the experimental error, which was found to be about 3%. The uncertainty (±*u*) was evaluated adopting 96% of the confidence level of the high speed data depending on the repeatability. The uncertainty (*σ_u_*) was [26]:

(1) $$\sigma_{u}=\sqrt{\int_{x_{o}}^{x_{n}}{\left(x-\mu_{e}\right)}^{2}p\left(x\right)dx}$$

where, *µ_e_* is the mean of *x* (measured data for droplet sliding velocity), *n* being the number of data points, and *p*(*x*) represents the probability distribution function. The diameter of the probability distribution was obtained via fitting the measured data in a Gaussian-function. The final result was normalized by the number of pixels, which contribute to the cross-correlation-peak. The bias uncertainty was evaluated as 0.04 pixels due to the complexities of assessing the low dimensional peaks in the distribution function. Hence, the uncertainty was evaluated as 3%.

Dust was collected from the local environment (Dammam in Saudi Arabia). The soft brushes were used to collect the dust particles from PV panel surfaces. The dust particles were later stored in the airtight containers before experiments. The size distribution of the dust particles was evaluated using a particle size analyzer. The particle shape and elemental composition of dust were characterized by using a scanning electron microscope, energy dispersive spectroscopy, and X-ray diffraction.

## 3. Results and Discussion

### 3.1. Crystallized Polycarbonate Surface and Dust Properties

**Figure 1:**
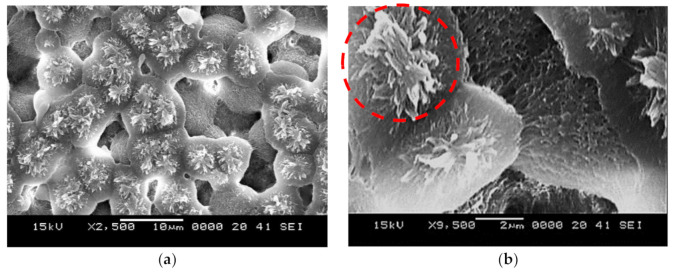
SEM images of crystallized surface: (**a**) micro size spherules, which represent the crystallized structures. Increasing acetone concentration or immersion duration, micro-size spherules become congested while lowering surface contact angle considerably and (**b**) fibrils on spherules (in the red dotted circle). Fibrils are formed from the crystallized sites and they create the Lotus effect reducing contact angle hysteresis.

**Figure 2:**
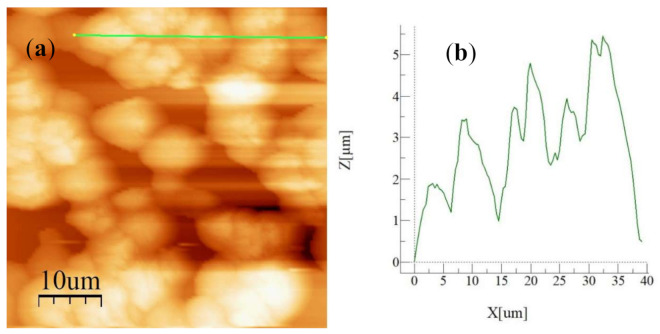
AFM (Atomic Force Microscope) surface image and line scan: (**a**) micro-image of the surface. The small bright spots represent the spherules and brightness is related to the height of the spherules (brighter the spot corresponds to higher the spherules), and (**b**) line scan, which is taken over the crystallized surface. It is shown as a green line in Figure 2a. It provides information on the height of the spherules.

**Figure 3:**
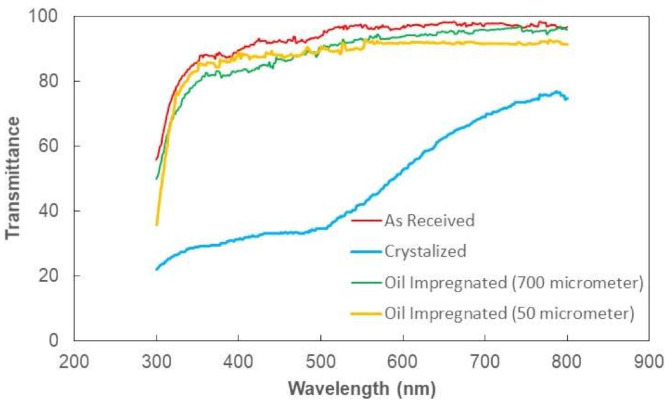
Optical transmittance of untreated, crystallized, and crystallized-oil impregnated polycarbonate. Spherules and fibrils on the crystallized surface cause diffuse reflection and partial absorption of incident UV visible radiation while lowering specular transmittance (wavelength-dependent transmittance). The increasing wavelength of incident optical radiation lowers diffuse reflection and absorption from the crystallized surface.

The solution treatment (30% concentration acetone) was used to crystallized polycarbonate surfaces via immersing technique. Figure 1a,b show top views of crystallized surfaces. The crystallized surface had a texture, which consisted of globules (Figure 1a) and fibers like whiskers (Figure 1b) emanating from the surface of the globules. The formation of whiskers was related to the secondary branching from the initially formed crystal sites [27]. The whiskers acted like nano-pins over the textured surface and contributed to the mobility of the liquid droplet, i.e., it created a Lotus effect on the surface. The size of the globules varies on the textured surface; however, they created a hierarchical texture morphology. Figure 2a,b show the surface image (Figure 2a) and texture profile (Figure 2b) of the crystallized surface obtained from an atomic force microscope. The globules on the surface were apparent from the profile peaks. The average roughness of the surface 3.2 µm and the surface roughness parameter (ratio of area covered by globulus over the projected area) was about 0.56. The wetting state of the surface before and after the crystallization was evaluated using a goniometer) via measuring contact angle and hysteresis. The contact angle of water was measured as 132° ± 4° on the crystallized surface while the hysteresis was 38° ± 5°. Hence, the crystallized surface demonstrated hydrophobic behavior; however, droplets adhered on the surface due to high hysteresis. To evaluate the opaqueness of the textured samples, optical transmittance measurement was carried out. Figure 3 shows optical transmittance of the textured and as received polycarbonate surfaces. The transmittance of the crystallized sample reduced significantly because of the texture; in which case, incident electromagnetic radiation diffused and scattered at the crystallized surface. In this case, spherules and fibrils on the crystallized surface caused diffuse reflection and partial absorption of incident UV visible radiation while resulting in scattering of incident ration and lowering specular transmittance (wavelength-dependent transmittance). The increasing wavelength of incident optical radiation lowered diffuse reflection and absorption from the crystallized surface. To enhance optical transmittance, the surface was impregnated with silicon oil. To cover the surface with a complete oil film, spreading factor of silicon oil on crystallized polycarbonate surface ($S_{o-pc}$) became important. The oil spreading is related to the surface tension of oil ($\gamma_{o}$), interfacial-shear at the oil-crystallized surface interface ($\gamma_{pc-o}$), and surface free energy of crystallized surface ($\gamma_{pc}$). This yields [11,28]: $S_{o-pc}=\gamma_{pc}-\gamma_{pc-o}-\gamma_{o}$. The surface free energy of the crystallized sample was evaluated in the early work, which is $\gamma_{pc}≅$ 36.2 mJ/m^2^ [24] and the surface tension of silicon oil is 0.0187 N/m [29]. The interfacial tension between oil and the crystalized surface could be evaluated through the Hemi–Wicking condition [30], i.e., it became: $\gamma_{pc-o}=\gamma_{s}-\frac{\gamma_{o}cos\theta_{pc-o}}{r}$, here, $cos\theta_{pc-o}$ is the droplet contact angle on the crystallized sample. The interfacial tension ($\gamma_{pc-o}$) was estimated as 4.3 mN/m. Hence, the spreading factor ($S_{o-pc}$) for silicon oil on the crystallized surface became 13.2 mN/m, which was greater than zero. This demonstrates that silicon oil spread over the crystallized surface. Hence, the silicon oil was spread over the crystallized surface under the controlled laboratory environment and, later, the oil film thickness was evaluated. Two extreme film thicknesses, 50 µm and 700 µm, were considered in the experiments. It is worth mentioning that as the oil film thickness reduced beyond 50 µm on the crystallized surface, the continuation of the oil film was not observed; oil formed islands like wet regions on the surface rather than developing a continuous film.

**Figure 4:**
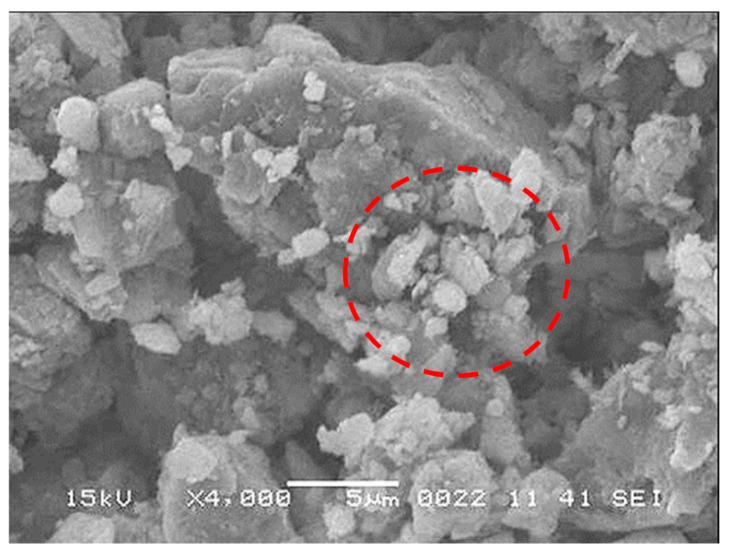
SEM Micro-image of dust particles. Dust particles have various shapes and sizes. Small dust particles adhere to large size particles forming clustering like structures as shown in the red dotted circle. The attachment of small particles demonstrates that these particles have increased polar forces.

The dust particles have varying shapes and sizes, and the average dust particle was assessed via particle analyzer (Malvern Panalytical, Mastersizer 3000, Worcestershire, UK), which was about 1.2 µm. Figure 4 shows dust particles, which were composed of small and large sizes. Small particles adhered to the large particle surface, which was because of the non-stoichiometric composition for the compounds (NaCl and KCl) formed in the dust. This is noted from Table 2, in which elemental constitutes of dust is given. The dust particles were composed of inorganic compounds. For small dust particles (≤2.5 µm), the stoichiometric ratio of elements for NaCl and KCl did not satisfy, which in turn created charges on the particles while contributing to the attachment of small particles to the large ones (Figure 4). The small size dust particles could adhere on the surface the charges and extra efforts were need for mitigating the dust from the dry crystallized sample surface. Therefore, the use of silicon oil may reduce the dust particle adhesion over the crystallized surface and sliding droplet can be able to pick up the dust from the oil film surface. The optical transmittance of the oil-impregnated surface was reassessed and findings are shown in Figure 3. The optical transmittance of the crystallized surface enhanced notably after oil spreading; however, the influence of the oil film thickness (700 µm and 50 µm) was not significant on the optical transmittance, i.e., optical transmittance remains high for both impregnated surfaces.

### 3.2. Dust Removal from Oil Surface and Droplet Dynamics

**Figure 5:**
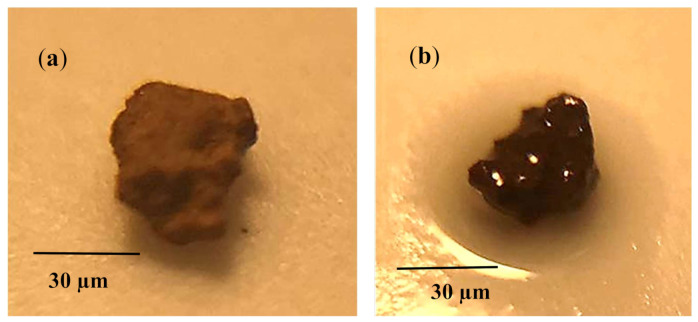
Optical image of dust particle: (**a**) before oil infusion, and (**b**) after oil infusion. Infused oil covers the dust particle surface, which indicates the positive spreading coefficient of oil over the particle surface.

**Figure 6:**
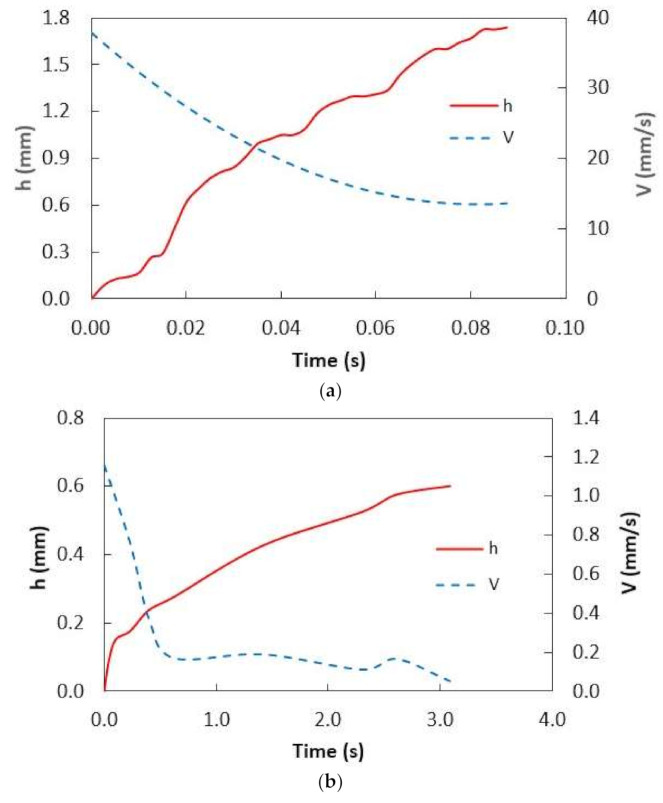
Infusion height and infusion velocity with time: (**a**) water, and (**b**) silicon oil. h represents infusion height on dust surface and V is the infusion velocity.

Mitigating dust from the oil film by a sliding water droplet is challenging because of the oil and water infusions (cloaking) on the dust particle. It is worth mentioning that a water droplet can cloak over the dust particle surface during sliding transition over the oil surface. In order to determine the liquid (water and oil) infusion velocity over the dust particles, many experimental tests are conducted and temporal progression of water and oil height over the dust particle surface is recorded via the high speed facility. Figure 5a,b show the optical images of the stationary dust particle prior to and after oil infusion with time, respectively, while Figure 6 shows the infusion (cloaking) velocity of water and silicon oil on the stationary dust particle. It is worth mentioning that the tracker program was used to obtain infusion velocity from the high-speed data. Infusion height of the water over the particle (~30 µm) was larger than that of silicon oil infusion height in the early period, i.e., water infusion took place at a faster rate than that of silicon oil. This resulted in larger water infusion velocity over the dust surface than that corresponding to silicon oil. As time progressed, the water and oil infusion velocities reduced and they became almost the same (silicon oil and water). Moreover, the decay of the infusion velocity was associated with the time, i.e., $V_{inf}\sim t^{-n}$, here Vinf is the infusion velocity of the liquid and it takes the value of about 1/4 [31]. Although silicon oil and water could spread over the dust particle surface, because of positive spreading coefficient (S), energy dissipation, due to viscous effect on the particle surface and gravitational pull over the particle height, slows down the liquid infusion on the surface [31]. Liquid infusion over dust surface was associated with the Joos’ law [32]. The liquid energy used during the infusion was related to the Ohnesorge number ($Oh=\mu /\sqrt{\rho a\gamma_{L}}$), where a represents the particle size [33]. The Ohnesorge number took different values for water and silicon oil because of density and viscosity of the fluids, i.e., density of water was 1000 kg/m^3^, viscosity was 0.7644 × 10^−3^ Pa.s, and surface tension was 72.3 mN/m while the density of silicon oil was 935 kg/m^3^, viscosity was 0.92 × 10^−2^ Pa, and surface tension was 35 mN/m. Hence, the Ohnesorge number took the values of 0.082 and 0.016 for water infusion on the dust particle sizes of 1.2 µm and 30 µm, respectively while it took values of 1.47 and 2.94 for silicon oil for the same sizes dust particles. Hence, energy dissipated by the silicon oil became larger than water during the complete infusion (cloaking) over the dust particle surface. This became more evident for the large size dust particle. Hence, the temporal behavior of the infusion height of the silicon oil over the dust particle surface became gradual as compared to that of water.

**Figure 7:**
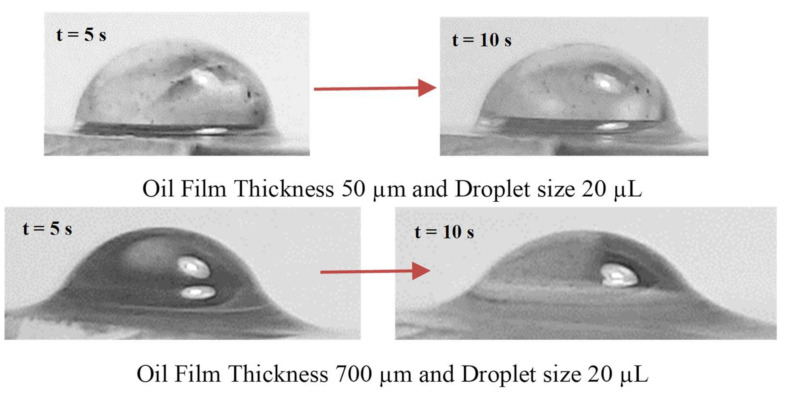
Optical image of silicon oil infusion on water droplet at different times. The time of droplet motion is started and recorded as the droplet is dispensed on the oil-impregnated sample surface.

Once the liquid droplet was created on the surface, droplet height reduced because of the bulging of droplet volume under gravity while causing droplet puddling on the surface. Once a water droplet was formed on the oil surface, a similar response was observed; however, silicon oil spread over the water droplet surface due to the lower surface tension of silicon oil as compared to water. It is worth mentioning that the interfacial resistance between oil and water was lower as compared to water surface tension. This caused oil infusion over the water droplet surface. Figure 7 depicts images of the droplet over the thin (50 µm) and thick oil (700 µm) films at which the oil ridge was formed around the water droplet rim because of oil infusion over the water droplet surface, particularly for large oil film thickness (700 µm). The oil ridge height was about ~0.32 mm around the droplet rim (three-phase-contact-line) for 700 µm thick oil film; however, it became ~0.09 mm for 50 µm thick oil film. Hence, thinning the oil film thickness reduced the ridge height around the droplet. The water droplet free surface (exposed to air) could be approximated by a spherical cap with a radius of $r_{o}=\sqrt{\left(2r_{d}-h_{o}\right)h_{o}}$, here ho is the height of the droplet cap in the air, r_o_ being the radius of the cap at the air-oil-water-contact-line, and rd is the droplet radius when droplet cap height was extended to form a circle [26]. The forces could be encountered due to the droplet pinning under interfacial shear across the boundary of water and oil. The pinning forces are formulated in the early work [26]; however, they are briskly described herein. The force equilibrium for the droplet, which is partially floating in the oil film, becomes: $F_{\gamma }\sin\left(\theta_{c}+\alpha \right)+F_{B}-W=0$, here $F_{\gamma }$ is the surface tension force, $F_{B}$ being the buoyancy force, and W represents the weight of the water droplet. Moreover, *α* being the water contact angle at the rim (air-water-oil-contact-line) and $\theta_{c}$ is the filling angle of the rim about the vertical axis. Rearrangement of the vertical force balance yields; $2r_{o}\gamma_{w-si}\sin\left(\theta_{c}+\alpha \right)-\frac{h_{o}^{2}}{3}\left(3r_{d}-h_{o}\right)\rho_{Si}g=0$, here $\gamma_{w-si}$ is water–silicon oil interface tension, $\rho_{Si}$ is silicon oil density, and *g* is gravity. The experimental data demonstrate that *h_o_* ≅ 0.92 mm for 20 µL droplet, which agrees with the value estimated from the vertical force balance (*h_o_* ≅ 1.02 mm). Moreover, as the oil-impregnated surface is inclined, both the water droplet and the oil film flow in the direction of gravity. The state of the film velocity ($\frac{\partial h_{f}}{\partial t}$) on the tilted sample with the tilt angle of *δ* ≤ π/2 is connected to the capillary number, $Ca=\frac{\mu_{s}u}{\gamma_{s}}$, where u is the velocity of the oil film and µS viscosity of oil [34]. The liquid velocity of the film on the tilted surface is formulated in the early study [34] and it takes the form:

$\frac{\partial h_{f}}{\partial t}=\nabla \cdot \left[h^{3}\nabla \nabla^{2}h_{f}\right]-L_{D}\nabla \cdot \left[h^{3}\nabla h_{f}\right]+\frac{\partial h_{f}^{3}}{\partial x}=0$, where *h_f_* is the normalized film thickness ($\bar{h_{f}}/h_{c}$, here $\bar{h_{f}}$ are the oil thickness and *h_c_* being the oil in the upstream of the film—maximum thickness), *x* represents the space along the inclined surface, and LD is the normalized parameter ($L_{D}={\left(3Ca\right)}^{1/3}cot\delta$, here *δ* is the tilt angle of the surface). The numerical solution of the equation for the film velocity yielded that the film velocity was 0.02 mm/s for the film thickness 700 µm and the tilt angle of 5°. The film velocity obtained by a tracker program using the high-speed recorded data was about 0.017 mm/s, which is closer to the numerical prediction. Moreover, since the crystallized surface, where the oil film was deposited, had a hydrophobic texture with 3.2 µm average roughness, slip condition could occur across the textured surface and the oil film. The slip velocity can be formulated through the slip length [35] ($b\sim \frac{\mu }{\mu_{o}}\left(1-\phi \right)h_{t}$, here μ being the droplet fluid viscosity, *ϕ* represents the solid fraction for crystallized surface, and *h_t_* is the film thickness) [30]. The solid fraction of the crystalized surface was evaluated via AFM and SEM micrographs and it was estimated at about *ϕ* = 0.35. Incorporating the viscosity of silicon oil (0.92 × 10^−2^ Pa.s) and water (0.89 × 10^−3^ Pa.s), film thickness (700 µm), and a solid fraction, the slip length yielded about *b* = 44 µm. Moreover, the slip velocity can be written as; $u_{s}=\frac{b}{\mu }\tau_{o-s}$, here $\tau_{o-s}$ is the interfacial shear stress across the oil and the crystallized surface [30], which can be further simplified after considering a Couette flow in clearance between the bottom of the droplet and oil. Hence, the slip velocity in terms of film velocity and the slip length yielded: $u_{s}\sim \frac{b}{h_{t}}u_{f}$. Moreover, the velocities of the partially immersed droplet and the oil film were different, which created a shear resistance across the droplet and the oil. The frictional stress can be; $\sim \mu \frac{\left(V_{d}-u_{f}\right)}{l_{m}}$, here $V_{d}$ represents the velocity of the droplet and *l_m_* is the height between the droplet base and the droplet mass center [36]. It is also known that shear stress in between the textured surface and the oil can be approximated as $\sim \mu_{o}\frac{\left(u_{f}-u_{s}\right)}{h_{t}}$. The shear stresses on the droplet and the oil at the interface had similar orders (stress continuity). This yields the relation between the slip and the droplet velocities, i.e., $\frac{V_{d}}{u_{f}}\sim 1+\frac{\mu_{o}}{\mu }\frac{l_{m}}{h_{t}}\left(1-\frac{b}{h_{t}}\right)$. The ratio of *b*/*h_t_* is about 0.057 and $\frac{l_{m}}{h_{t}}\gg 1$, which yields that the droplet velocity becomes much larger than the film velocity ($V_{d}$ > *u_f_*). Moreover, energy dissipated by the sliding water droplet on the oil film via shear resistance was related to (i) shear rate created at the ridges due to oil infusion over the droplet surface (ii) the air drag acting on the droplet cap in the air, (iii) lateral component of the surface tension around the droplet rim. The dissipated energy is formulated in the early study [30] and it takes the form ~Δ*L*($\mu \left(\frac{V_{d}-u_{f}}{l_{m}}\right)\pi r_{o}{}^{2}+\mu_{o}\left(V_{d}-u_{f}\right)2\pi r_{o}+6\pi \mu_{o}h_{o}\left(V_{d}-u_{f}\right)+C_{d}\rho_{a}A_{c}V_{d}^{2}$). Here, the term Δ*L* is an incremental length scale along the oil film surface, $\mu_{o}\left(V_{d}-u_{f}\right)2\pi r_{o}$ represents the interfacial shear effect on the immersed droplet base [36], $\mu_{o}\left(V_{d}-u_{f}\right)2\pi r_{o}$ is the shear effect due to ridge height around the droplet meniscus [30], $6\pi \mu_{o}h_{o}\left(V_{d}-u_{f}\right)$ is the force of drag force created over the immersed droplet by the oil [37], where *h_o_* is the droplet immersion height in oil, which is experimentally evaluated to be ~62 µm, and $C_{d}\rho_{a}A_{c}{\left(V_{d}-u_{f}\right)}^{2}$ is the air drag acting on the droplet cap, where *C_d_* is the drag coefficient, and *A_c_* is the cross-sectional area of the droplet cap in the air. In addition, work was done by the sliding droplet towards overcoming the droplet pinning, which could account for the droplet’s additional energy dissipation. The work required overcoming the pinning was about $2\pi \gamma_{w-Si}r_{o}cos\left(\theta_{c}+\alpha \right)∆L$ [26]. The potential and kinetic energy change along the incremental length scale were $\sim m_{d}g∆Lsin\delta$ and is $\frac{1}{2}m_{d}{\left(V_{d}-u_{f}\right)}^{2}$, respectively. Here, δ is the inclination angle of the surface and *m_d_* is the mass of the droplet ($\rho_{w}\frac{4\pi }{3}r_{o}^{3}$, *r_o_* being the droplet radius when the droplet volume is spherical). Hence, the change of droplet potential energy along the incremental length scale overcame the frictional energy dissipation and the work required for pinning, and, in addition, the kinetic energy for creating the droplet motion on the oil surface. The potential energy change of the droplet per unit droplet mass along the length scale of 44 mm of 10° inclined surface with 700 µm thick oil was 0.075 J/kg while the kinetic energy was 3.125 × 10^−3^ J/kg. Hence, a large amount of energy was dissipated in the course of droplet sliding, i.e., almost 96% of droplet potential energy was dissipated to overcome shear resistance and pinning of the droplet.

**Figure 8:**
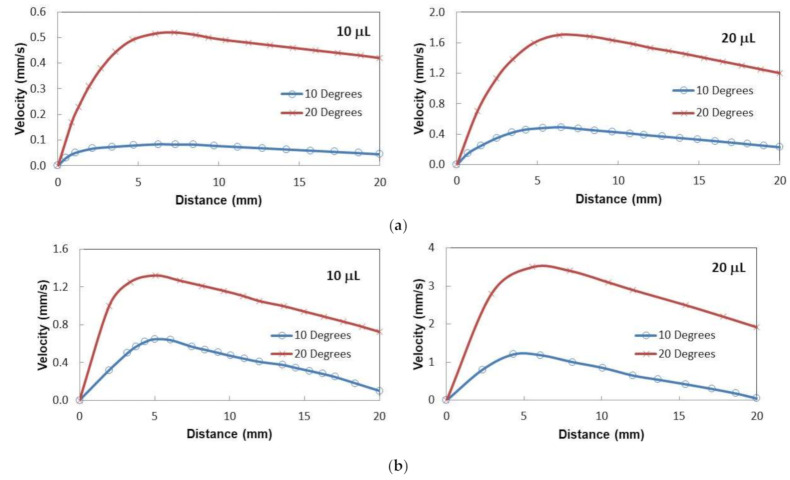
Droplet sliding velocity on inclined oil impregnated surface for two droplet volume and inclination angles. (**a**) Oil film thickness is 50 µm. (**b**) Oil film thickness is 700 µm.

Figure 8a shows droplet velocity on the thin oil film (50 µm thick) with distance for two volumes and two tilt angles. In the early period of sliding, the velocity increased to attain maximum and as the droplet sliding continued along with the inclined film, the velocity decreased gradually. This behavior was observed for both inclination angles and the droplet volumes. This is mainly related to the oil ridge formation in the droplet neighborhood through oil infusion on the water droplet. Oil infusion in the early sliding period was small preventing the oil ridge formation. As the droplet sliding progressed, the oil ridge formed around the droplet because of the oil infusion during the droplet sliding. Hence, the interfacial fluid resistance between the oil and the droplet liquid increased along the droplet circumference, which caused slowing down the droplet motion along the inclined surface. Moreover, as the droplet size increased, interfacial resistance along the droplet ridge increased because of the increased droplet diameter with increasing droplet volume. However, increased droplet inertial force, because of the droplet mass increase, enhanced the droplet velocity despite the fact that interfacial resistance at oil-droplet along with the ridge increased. In the case of the thick silicon oil film (~700 µm) impregnated sample surface, the sliding velocity of the droplet increased to reach its maximum and, later, it decreased gradually along the inclined surface (Figure 8b). This behavior was similar to the thin oil film case (Figure 8a). The sliding velocity attains larger values for the thick oil film case than that of the thin film case. It is worth noting that the droplet immersion became more as the film thickness becomes larger (~700 µm). Hence, it was expected that the fluid resistance around the immersed area of the droplet would increase. However, the attainment of the large velocity on the thick oil film suggests that the droplet on the thin film (~50 µm) penetrated into the oil film and making physical contact on the crystallized surface. This increased the shear resistance at the droplet bottom. In addition, the droplet located on the thick oil film floated in the oil film and created slip condition at the droplet bottom, which in turn reduced the shear resistance across the film and the droplet. Therefore, the velocity of the droplet attained higher values for the large thickness oil-impregnated sample surface.

**Figure 9:**
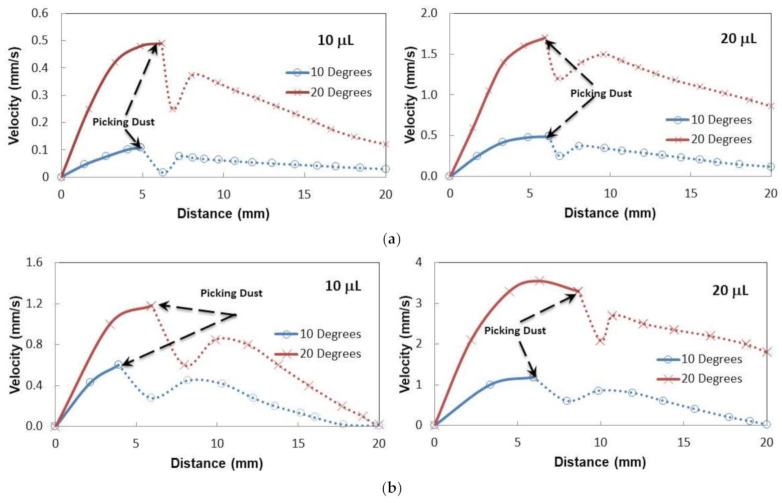
(**a**) Droplet sliding velocity on inclined oil impregnated surface with the large dust particle presence for two droplet volume and tilting angles. Oil film thickness is 50 µm. (**b**) Droplet sliding velocity on inclined oil impregnated surface with large dust particle presence for two droplet volume and tilting angles. Oil film thickness is 700 µm.

**Figure 10:**
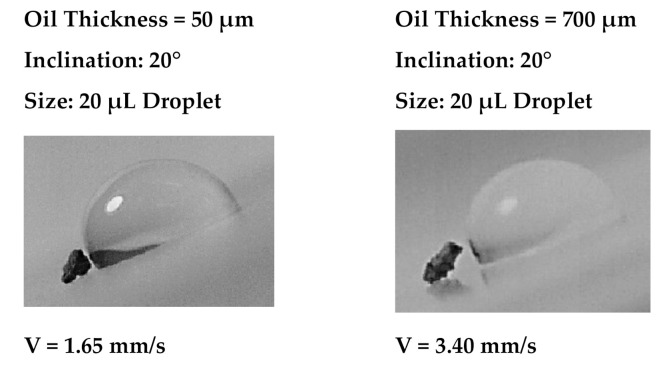

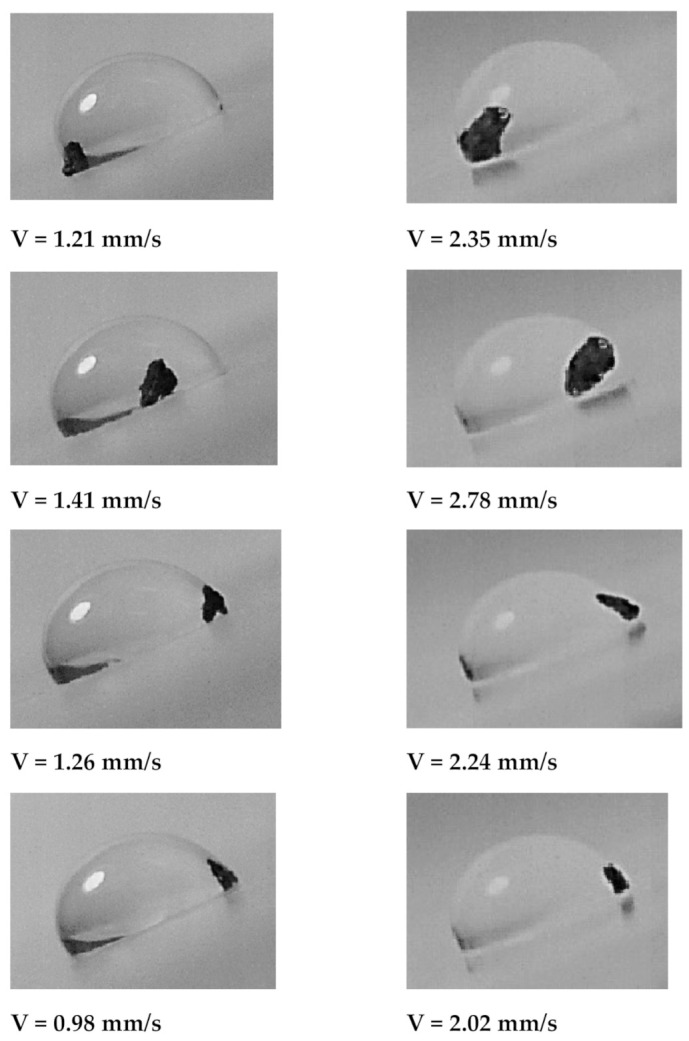
Stages of the droplet on inclined oil film for two oil film thicknesses. The dark color represents the dust particle.

Figure 9a,b shows droplet sliding velocity on the inclined thin and thick oil films (~50 µm and 700 µm) with the presence of the dust particle over the oil surface for two droplet volumes and two tilting angles of the sample. Figure 10 depicts the optical images of different stages of the dust particle mitigated by the sliding water droplet on the inclined oil film. It is worth mentioning that the dust particle size selected was large enough to be recorded by a high speed facility; hence, the selected dust particle was about 75 µm. As the sliding droplet approached the vicinity of the dust particle, the oil ridge around the droplet first contacted the dust particle surface. However, the dust particle height was larger than the oil ridge height, the infusion of oil from the oil ridge towards the dust particle surface was expected to be not considerable. In addition, the contact duration between the oil ridge and the dust particle was short, which reduced the amount of oil-infused over to the dust particle surface. Moreover, once the droplet fluid wetted the dust particle on the oil surface, droplet sliding velocity reduced, and the part of the dust particle surface, which was not infused by the oil, was infused by the fluid of the droplet. In the course of fluid infusion over the particle surface, the sliding velocity of the droplet reduced further. Once the droplet liquid cloaked over the dust, the particle was picked up by the droplet fluid and the particle was reoriented inside the sliding droplet, which can be observed from Figure 10. During the dust particle reorientation, the sliding droplet mass increased slightly and the inertia force increases while causing droplet sliding velocity to increase along the inclined surface. However, as the dust particle was oriented at the droplet trailing edge, which was opposing to the droplet front edge in the sliding direction, the droplet velocity reduced considerably. This situation can be observed in Figure 10. Moreover, it became true for all volumes of the droplet, the tilting angle of the sample, and oil film thickness. The possible explanation of this behavior is that the dust particle in the droplet trailing edge immersed into the oil and it was anchored by the oil film. This created the pinning influence on the sliding droplet, which gradually ceased sliding on the oil film, i.e., sliding motion terminated and the sliding velocity became zero on the oil film. The inclination angle had a significant influence on the droplet sliding velocity. In this case, reducing the inclination angle from 20° to 10° lowered the sliding speed from 0.98 mm/s to 2.02 mm/s. For a small inclination angle (10°), the dust particle position in the sliding droplet had a considerable effect on the sliding velocity. In this case, droplet sliding velocity attained high values as the dust particle was located in the central region of the droplet. As the dust particles moved towards the trailing edge of the droplet, the sliding velocity reduced considerably. Hence, the dust particles partially became immersed in the oil film at the droplet edge while creating the anchoring effect on the droplet sliding velocity.

## 4. Conclusions

The sliding motion of a water droplet over silicon oil-impregnated sample is investigated. The influence of the tilting angle of the surface, oil film thickness, and droplet size on the droplet sliding behavior is analyzed. Polycarbonate surface is solution crystallized, using 30% concentrated acetone solution, to create surface texture resulting in the hydrophobic wetting state. The solution crystallization of the surface gives rise to low optical transmittance of the samples. Silicon oil impregnation improves the optical transmittance of the samples and enables water droplets to slide over the surface. Impregnated oil film thickness influences the sliding velocity of the droplet; hence, the sliding velocity becomes lower for the small thickness oil film (~50 µm) than that corresponding to the large oil film thickness (~700 µm). This is related to the interfacial resistance at the droplet bottom, i.e., slip velocity at the interface across the oil film and droplet fluid becomes small and it is also likely that the droplet bottom becomes in contact with the crystallized surface (touching) rather than floating in the oil film. As the film thickness increases, the droplet floats within the oil film, which increases the sliding velocity over the film. The oil ridges formed over the circumference of the droplet contribute adversely to the sliding over the inclined oil film due to fluid resistance occurring at the inter-boundary of the droplet fluid and the oil around the ridge sites. The dust particle located on the oil film is partially cloaked by the oil, which makes it difficult to remove by the sliding droplet. Nevertheless, the sliding droplet cloaks and removes the particle from the oil film. Once the dust particle is removed by the droplet fluid, the orientation of the dust particle inside the droplet fluid changes during the sliding. This causes the first reduction and later enhancing the velocity of the droplet over the film. As the dust particle picked up settles in the trailing edge of the sliding droplet, the sliding velocity decreases gradually. In this case, the particle in the trailing edge of the droplet is partially immersed in the oil film while creating a pinning effect on the sliding droplet. Nevertheless, the pinning influence of the dust particle does not cease the droplet motion on the surface for thin oil film (50 µm); however, the droplet sliding ceases for the thick oil film (µm). The present study provides a detailed analysis of water droplet sliding behavior on the inclined oil-impregnated crystallized polycarbonate surface and explores the water droplet sliding conditions and environmental dust mitigation from the inclined oil-impregnated surface.

## Figures and Tables

**Table 1:** Parameters used in solution crystallization and dust mitigation from oil impregnated surfaces. In the experiments, four concentrations of acetone solution and four levels of immersion are considered during crystallization of polycarbonate surface. The acetone concentration and immersion duration maximizing water contact angle (132° ± 4°) are selected, which are 30% acetone concentration 4 min of immersion duration.

Source of Parameters	
Acetone Concentration for Solution Crystallization (%volume)	30 to 60
Immersion Duration for Solution Crystallization (s)	60 to 480
Impregnated Oil Film Thickness (µm)	50 and 700
Water Droplet Volume (µL)	10 to 20
Inclination Angle of Oil Impregnated surface (Degrees)	10 to 20

**Table 2:** Elemental constitute of dust particles (wt%). Increasing dust particle size causes a reduction in alkaline earth metal (Ca) and silicon (Si); however, alkaline metals (Na and K) and chlorine (Cl).

Size	Si	Ca	Na	S	Mg	K	Fe	Cl	O
**≥1.2 µm**	11.6	8.5	2.1	1.1	2.5	0.8	1.2	0.4	Balance
**<1.2 µm**	10.4	7.7	2.5	2.1	1.8	1.2	1.1	1.1	Balance

## Data Availability

The data associated with the current work are available upon request from the corresponding author.

## Sample Availability

Samples of the compounds are available from the authors upon requests.

## References

[1] Yilbas B.S., Al-Qahtani H., Al-Sharafi A., Bahattab S., Hassan G., Al-Aqeeli N., Kassas M. (2019). Environmental Dust particles Repelling from A Hydrophobic surface under electrostatic Influence. Sci. Rep..

[2] Bergin M.H., Ghoroi C., Dixit D., Schauer J.J., Shindell D.T. (2017). Large reductions in solar energy production due to dust and particulate air pollution. Environ. Sci. Technol. Lett..

[3] Mcguire C. (2019). Dust to dust. Lancet. Respir. Med..

[4] Taheri F., Forouzani M., Yazdanpanah M., Ajili A. (2020). How farmers perceive the impact of dust phenomenon on agricultural production activities: A Q-methodology study. J. Arid Environ..

[5] Farfan J., Breyer C. (2018). Combining floating solar photovoltaic power plants and hydropower reservoirs: A virtual battery of great global potential. Energy Procedia.

[6] Chanchangi Y.N., Ghosh A., Sundaram S., Mallick T.K. (2020). Dust and PV performance in Nigeria: A review. Renew. Sustain. Energy Rev..

[7] Al Shehri A., Parrott B., Carrasco P., Al Saiari H., Taie I. (2016). Impact of dust deposition and brush-based dry cleaning on glass transmittance for PV modules applications. Sol. Energy.

[8] Xu Q., Zhang W., Dong C., Sreeprasad T.S., Xia Z. (2016). Biomimetic self-cleaning surfaces: Synthesis, mechanism and applications. J. R. Soc. Interface.

[9] Adukwu J.E., Yilbas B.S., Jalilov A.S., Al-Qahtani H., Yaqubu M., Abubakar A.A., Khaled M. (2020). Adhesion characteristics of solution treated environmental dust. Sci. Rep..

[10] Smith J.D., Dhiman R., Anand S., Reza-Garduno E., Cohen R.E., McKinley G.H., Varanasi K.K. (2013). Droplet mobility on lubricant-impregnated surfaces. Soft Matter.

[11] Anand S., Rykaczewski K., Subramanyam S.B., Beysens D., Varanasi K.K. (2015). How droplets nucleate and grow on liquids and liquid impregnated surfaces. Soft Matter.

[12] Malavasi I., Bernagozzi I., Antonini C., Marengo M. (2015). Assessing durability of superhydrophobic surfaces. Surf. Innov..

[13] Huang Z., Gurney R.S., Wang T., Liu D. (2018). Environmentally durable superhydrophobic surfaces with robust photocatalytic self-cleaning and self-healing properties prepared via versatile film deposition methods. J. Colloid Interface Sci..

[14] Eshaghi A. (2020). Fabrication of transparent silica-silica nanotube/PFTS nano-composite thin films with superhydrophobic, oleophobic, self-cleaning, and anti-icing properties. Opt. Quantum Electron..

[15] Zhong Y., Gu L., Wang S., Jin Y., Xiao H. (2019). Green and superhydrophobic coatings based on tailor-modified lignocellulose nanofibrils for self-cleaning surfaces. Ind. Eng. Chem. Res..

[16] Fenero M., Palenzuela J., Azpitarte I., Knez M., Rodríguez J., Tena-Zaera R. (2017). Laponite-based surfaces with holistic self-cleaning functionality by combining antistatics and omniphobicity. Acs Appl. Mater. Interfaces.

[17] Qi J., Wang L., Yan F., Xue Q. (2013). The tribological performance of DLC-based coating under the solid-liquid lubrication system with sand-dust particles. Wear.

[18] Sousa M.F.B., Barbosa G.F., Signorelli F., Bertran C.A. (2017). Anti-scaling properties of a SLIPS material prepared by silicon oil infusion in porous polyaniline obtained by electropolymerization. Surf. Coat. Technol..

[19] Rifai A., Abu-Dheir N., Khaled M., Al-Aqeeli N., Yilbas B.S. (2017). Characteristics of oil impregnated hydrophobic glass surfaces in relation to self-cleaning of environmental dust particles. Sol. Energy Mater. Sol. Cells.

[20] Figgis B., Guo B., Javed W., Ahzi S., Rémond Y. (2018). Dominant environmental parameters for dust deposition and resuspension in desert climates. Aerosol Sci. Technol..

[21] Yilbas B.S., Ali H., Al-Aqeeli N., Khaled M.M., Said S., Abu-Dheir N., Merah N., Youcef-Toumi K., Varanasi K.K. (2016). Characterization of environmental dust in the Dammam area and mud after-effects on bisphenol-A polycarbonate sheets. Sci. Rep..

[22] Ferrari M., Cirisano F. (2020). High transmittance and highly amphiphobic coatings for environmental protection of solar panels. Adv. Colloid Interface Sci..

[23] Yilbas B.S., Al-Sharafi A., Ali H., Al-Aqeeli N., Al-Qahtani H., Al-Sulaiman F., Abu-Dheir N., Abdelmagid G., Elkhazraji A. (2018). Environmental dust removal from inclined hydrophobic glass surface: Avalanche influence on dynamics of dust particles. RSC Adv..

[24] Aharoni S.M., Murthy N.S. (1998). Effects of solvent-induced crystallization on the amorphous phase of polycarbonate of bisphenol A. Int. J. Polym. Mater..

[25] Heib F., Schmitt M. (2016). Statistical Contact Angle Analyses with the High-Precision Drop Shape Analysis (HPDSA) Approach: Basic Principles and Applications. Coatings.

[26] Armstrong S., McHale G., Ledesma-Aguilar R., Wells G.G. (2019). Pinning-free evaporation of sessile droplets of water from solid surfaces. Langmuir.

[27] Bhattacharya S., Charonko J.J., Vlachos P.P. (2018). Particle image velocimetry (PIV) uncertainty quantification using moment of correlation (MC) plane. Meas. Sci. Technol..

[28] Lauritzen J.I., Hoffman J.D. (1960). Theory of formation of polymer crystals with folded chains in dilute solution. J. Res. Natl. Bur. Stand. Sect. A Phys. Chem..

[29] Kim D., Pugno N.M., Ryu S. (2016). Wetting theory for small droplets on textured solid surfaces. Sci. Rep..

[30] Ricci E., Sangiorgi R., Passerone A. (1986). Density and surface tension of dioctylphthalate, silicone oil and their solutions. Surf. Coat. Technol..

[31] Phan C.M. (2014). Stability of a Floating Water Droplet on an Oil Surface. Langmuir.

[32] Hassan G., Yilbas B.S., Al-Qahtani H. (2020). Droplet fluid infusion into a dust layer in relation to self-cleaning. RSC Adv..

[33] Bergeron V., Langevin D. (1996). Monolayer spreading of polydimethylsiloxane oil on surfactant solutions. Phys. Rev. Lett..

[34] Carlson A., Kim P., Amberg G., Stone H.A. (2013). Short and long time drop dynamics on lubricated substrates. EPL Europhys. Lett..

[35] Schwartz L.W. (1989). Viscous flows down an inclined plane: Instability and finger formation. Phys. Fluids A Fluid Dyn..

[36] Ng C.-O., Wang C.Y. (2010). Apparent slip arising from Stokes shear flow over a bidimensional patterned surface. Microfluid. Nanofluidics.

[37] Yang S.-M. (1987). Motions of a sphere in a time-dependent stokes flow: A generalization of Faxen’s law. Korean J. Chem. Eng..

